# IRF7 Is Required for the Second Phase Interferon Induction during Influenza Virus Infection in Human Lung Epithelia

**DOI:** 10.3390/v12040377

**Published:** 2020-03-29

**Authors:** Wenxin Wu, Wei Zhang, Lili Tian, Brent R. Brown, Matthew S. Walters, Jordan P. Metcalf

**Affiliations:** 1 Pulmonary, Critical Care & Sleep Medicine, Department of Medicine, the University of Oklahoma Health Sciences Center, Oklahoma City, OK 73104, USA; 2 Department of Microbiology and Immunology, the University of Oklahoma Health Sciences Center, Oklahoma City, OK 73104, USA; 3 Veterans Affairs Medical Center, Oklahoma City, OK 73104, USA

**Keywords:** RIG-I, IRF3, IRF7, TLR3, interferon, influenza, A549, human, lung, innate immunity

## Abstract

Influenza A virus (IAV) infection is a major cause of morbidity and mortality. Retinoic acid-inducible protein I (RIG-I) plays an important role in the recognition of IAV in most cell types, and leads to the activation of interferon (IFN). We investigated mechanisms of RIG-I and IFN induction by IAV in the BCi-NS1.1 immortalized human airway basal cell line and in the A549 human alveolar epithelial cell line. We found that the basal expression levels of RIG-I and regulatory transcription factor (IRF) 7 were very low in BCi-NS1.1 cells. IAV infection induced robust RIG-I and IRF7, not IRF3, expression. siRNA against IRF7 and mitochondrial antiviral-signaling protein (MAVS), but not IRF3, significantly inhibited RIG-I mRNA expression and IFN induction by IAV infection. Most importantly, even without virus infection, IFN-β alone induced RIG-I, and siRNA against IRF7 did not inhibit RIG-I induction by IFN-β. Similar results were found in the alveolar basal epithelial A549 cell line. RIG-I and IRF7 expression in humans is highly inducible and greatly amplified by IFN produced from virus infected cells. IFN induction can be separated into two phases, that initially induced by the virus with basal RIG-I (the first phase), and that induced by the subsequent virus with amplified RIG-I from the first phase IFN (the second phase). The *de novo* synthesis of IRF7 is required for the second phase IFN induction during influenza virus infection in human lung bronchial and alveolar epithelial cells.

## 1. Introduction

Influenza A virus (IAV) infection is a major cause of morbidity and mortality. In 2009, a pandemic caused by the novel H1N1 IAV infected over 300,000 individuals with at least 16,000 confirmed deaths worldwide [1]. The 2017–2018 flu season has been the worst season in the US since 2009. It is estimated that the burden of illness during the 2017–2018 season was high with an estimated 45 million people getting sick with influenza, 21 million people going to a health care provider, 810,000 hospitalizations, and 61,000 deaths from influenza (from 2017–2018 Estimated Influenza Illnesses, Medical visits, Hospitalizations, and Deaths and Estimated Influenza Illnesses, Medical visits, Hospitalizations, and Deaths Averted by Vaccination in the United States, Centers for Disease Control and Prevention). 

The innate immune system recognizes and responds to pathogens in a non-specific manner and provides immediate protection against infection. Induction of interferon (IFN) is a critical component of the host innate immune response to influenza virus infection. IFNs are further divided into type I (mainly IFN-α and β), II (IFN-γ) and III (IFN-λ) subtypes, based in part on the differential use of unique receptors through which they mediate signal transduction to induce anti-viral activity [2]. The IFN responses to IAV are triggered by the recognition of pathogen-associated molecular patterns (PAMPs) by host internal or cell surface pattern recognition receptors (PRRs), including retinoic acid-inducible protein I (RIG-I) and Toll-like receptor 3 (TLR3) [3]. RIG-I is essential for IFN induction during RNA virus infections of non-plasmacytoid dendritic cell (pDC) cell types [4]. TLR3, a double-stranded RNA sensor, may be used by some epithelial cells to detect the viral replicative intermediate dsRNA [5]. Mitochondrial antiviral-signaling protein (MAVS), the intermediary protein of RIG-I, forms protease resistant prion-like aggregates that activate specific signaling pathways leading to activation of NF-κB and induction of the interferon regulatory transcription factors (IRF)3 and IRF7. NF-κB is crucial for inflammatory cytokine induction. IRF3 and IRF7 are then translocated into the nucleus where they act as transcription factors for the production of type I and III IFNs [6,7]. We have previously shown that both RIG-I and TLR3 are important for IFN induction by IAV in human lung alveolar epithelial cells (AEC) [3]. We also developed a human lung tissue model using precision-cut slices to study the local lung response to IAV, and we showed that RIG-I is critical for the initiation of the early antiviral cytokine response in the human lung [8].

IFN responses are the central component of the innate immune system’s control of viral infection [9]. Once released, type I and III IFNs bind to their respective receptors (IFNAR and IFNLR1/IL10R2, respectively) on the same and neighboring cells. When an IFN interacts with its cognate receptor, a signal is rapidly transmitted within the cell. The primary signal transduction cascade promoted by IFNs is mediated by the Janus family of protein tyrosine kinase 1 (JAK1) signal transducers and activators of the transcription (STAT) pathway [10]. Receptor engagement subsequently leads to the activation of the IFN-stimulated regulatory factor 3 (ISGF3) transcription complex. ISGF3 is composed of STAT1 and STAT2, both of which are activated by JAK1, and IRF9 [7]. The activation of this transcriptional activator complex leads to increased expression of interferon-stimulated genes (ISGs), including 2′,5′-oligoadenylate synthetase (OAS), Mx proteins, and protein kinase R (PKR), inducing an antiviral state [11]. RIG-I and IRF7 are also ISGs that are up-regulated and further amplify the entire antiviral immune system [12]. The basal expression level of RIG-I is very low in human lung. However, RIG-I expression is highly inducible and greatly amplified by first phase IFN production from the viral infected cells in an autocrine and paracrine fashion [13,14]. Therefore, there are two phases of IFN induction after IAV infection. The first phase IFN is induced when the viral genome is recognized by basal level RIG-I in the IAV-infected cells. The secreted first phase IFN proteins then bind to its cognate cellular receptor and induce vast amounts of RIG-I and IRFs in the same and neighboring cells. The significantly elevated RIG-I and IRF proteins turn the infected and uninfected neighboring cells into an “immunoactive” state primed to respond to subsequent viral RNA detection. Thus, second-phase IFN induction will be induced when IAV is detected in newly infected, or already infected, IFN-stimulated cells.

The role of IRF3 and IRF7 in the host response to IAV infection was analyzed in IRF3^−/−^, IRF7^−/−^, and IRF3^−/−^IRF7^−/−^ knockout mice [15]. While the absence of IRF3 had only a moderate impact on IFN expression, deletion of IRF7 completely abolished IFNα production after infection. In contrast, lack of both IRF3 and IRF7 resulted in the absence of both IFNα and IFNβ after IAV infection. Ciancanelli et al. reported that human IRF7 deficiency impaired IFN amplification in pDC, leukocytes, induced pluripotent stem cell (iPSC)-derived pulmonary epithelial cells [16]. Despite advances in the understanding of innate response to influenza in the mouse model, it is essential to conduct further studies in humans to decipher the innate immune responses to IAV, particularly at the site of infection [17]. Epithelial cells that line the conducting (bronchial) and respiratory (alveoli) airways are the primary site of IAV replication in the lung [18]. In response to viral infection, epithelial cells trigger the innate immune response to limit viral dissemination. To examine the role of IRF7 in amplified second phase induction of IFN during IAV infection of the human lung epithelium, we used immortalized cell lines representative of the bronchial (BCi-NS1.1, [19]) and alveolar (A549) lung epithelium.

## 2. Materials and Methods

### 2.1. Cell Culture

The BCi-NS1.1 cell line was derived from human airway basal cells via expression of a retrovirus expressing human telomerase and was a kind gift of Dr. Ronald Crystal, Cornell Medical Center [19]. BCi-NS1.1 cells were cultured in BEBM^TM^ Bronchial Epithelial Cell Growth Basal Medium (Lonza, cat#CC-3171) with SingleQuots^TM^ Supplements and Growth Factors (Lonza, cat#CC-4175). The human pulmonary epithelial cell line, A549, was obtained from the American Type Culture Collection (ATCC, Manassas, VA, CCL-185). A549 cells were propagated in Dulbecco’s modified Eagle Medium (DMEM) containing 10% fetal bovine serum (FBS), 2 mM L-glutamine, and 80 µg of gentamicin/mL.

### 2.2. Preparation of Influenza Virus Stock

H1N1 influenza virus, A/PR/34/8 (PR8), was passaged in Madin-Darby canine kidney (MDCK) cells. Virus was propagated in MDCK cells in DMEM/F12 with ITS+ (BD Biosciences, Franklin Lakes, NJ) and trypsin, harvested at 72 h post-infection and titered by plaque assay in MDCK cells. The virus was stored in aliquots at −80 °C. There was no detectable endotoxin in the final viral preparations used in the experiments as determined by limulus amebocyte lysate assay (Cambrex, Walkersville, MD). The lower limit of detection of this assay is 0.1 EU/mL or approximately 20 pg/mL LPS. 

### 2.3. siRNA Transfection into BCi-NS1.1 and A549 Cells

IRF7 siRNA (siIRF7) and MAVS siRNA (siMAVS) were purchased from Dharmacon (Cat# L-011810-00 and Cat# L-024237-00). IRF3 siRNA (siIRF3) and negative control siRNA were purchased from Ambion (Cat# AM16708A and Cat# 4390843). For siRNA treatment, cells were plated and cultured for 24 h before treating with siRNAs (40 nM). siRNA was diluted in 250 µL of Opti-MEM medium and mixed gently. Five µL of Lipofectamine 2000 (Invitrogen) was added to 250 µL Opti-MEM medium and incubated for 5 min. Diluted siRNA and Lipofectamine 2000 were combined and mixed gently and incubated for 20 min at RT. The siRNA-Lipofectamine 2000 complexes were added to each well and mixed gently. The siRNA final concentration was 100 nM. The cells were incubated at 37 °C for 24 h and fresh media were added to replace the siRNA containing transfection media.

### 2.4. Lactate Dehydrogenase (LDH) Assay 

Staurosporine was used as a positive control (Enzo Biochem Inc., Farmingdale, NY, USA). LDH activity was measured using a coupled enzymatic reaction using a commercially available kit (BioVision Incorporated, Milpitas, CA, USA). The amount of LDH activity was assessed by detection of the reaction product, formazan, at 500 nm using a spectrophotometer (Vmax Microplate reader, Molecular Devices, Sunnyvale, CA, USA). Cytotoxicity was expressed as the percentage of LDH released (supernatant) of the total LDH present (cell + supernatant).

### 2.5. Measurement of mRNA Expression by Quantitative Real-Time PCR (qRT-PCR) 

Total RNA from cells was extracted using a modified TRIzol (Invitrogen, Carlsbad, CA, USA) protocol, spectrophometrically quantitated, and the integrity was verified by formaldehyde agarose gel electrophoresis. RNA was treated with DNAse to remove genomic DNA contamination. Equal amounts (1 µg) of RNA from each sample were reverse-transcribed using oligo (dT) as primers for production of cDNA (SuperScript II First-Strand Synthesis System for RT-PCR, Invitrogen, Carlsbad, CA, USA). Gene specific primers for PRRs, cytokines and β-actin housekeeping genes were used. qRT-PCR was performed using SYBR Green (Quanta Biosciences, Gaithersburg, MD, USA) in a Bio-Rad CFX96TM Touch Real-Time PCR Detection System. Results were calculated and graphed from the ΔCT of the target gene and normalizing housekeeping gene control. The primers’ sequences were as follows: RIG-I forward 5′-TCCTTTATGAGTATGTGGGCA-3′; RIG-I reverse 5′-TCGGGCACAGAATATCTTTG-3′; IFN-β forward 5′-GCTCTCCTGTTGTGCTTCTCCAC-3′; IFN-β reverse 5′-CAATAGTCTCATTCCAGCCAGTGC-3′; β-actin forward 5′-GCCAACCGCGAGAAGATGACC-3′; β-actin reverse 5′-CTCCTTAATGTCACGCACGATTTC-3′; TLR3 forward 5′-GTCTGGGAACATTTCTCTTC-3′; TLR3 reverse 5′-GATTTAAACATTCCTCTTCGC-3′; IRF7 forward 5′-CAGATCCAGTCCCAACCAAG-3′; IRF7 reverse 5′-GTCTCTACTGCCCACCCGTA-3′; IFN-λ1 forward 5′-CGCCTTGGAAGAGTCACTCA-3′; IFN-λ1 reverse 5′-GAAGCCTCAGGTCCCAATTC-3′; IL-6 forward 5′-AGGAGCCCAGCTATGAACT-3′; IL-6 reverse 5′-TGAGATGCCGTCGAGGATG-3′; MAVS forward 5′-CAATGCCGTTTGCTGAAGAC-3′; MAVS reverse 5′-ATTCCTTGGGATGGCTCTGG-3′.

### 2.6. RIG-I, TLR3 and IRF7 Protein Determination by Immunoblotting

The cells were harvested and homogenized, and then lysed in 500 µL of cold lysis buffer (150 mM NaCl, 50 mM Tris, pH 8.0, 10 mM EDTA, NaF, sodium pyrophosphate, 1% NP-40, 0.5% sodium deoxycholate, 0.1% SDS, 10 µg of leupeptin/mL). Cell homogenates were clarified by centrifugation at 10,000× *g*, at 4 °C for 10 min, and the clarified lysates were mixed with SDS-PAGE sample buffer (60 mM Tris, pH 6.8, 10% glycerol, 2.3% SDS) and heated to 95 °C for 5 min. The samples were separated using a 4–15% gradient gel and electrophoretically transferred to polyvinylidene fluoride (PVDF) membranes. For the detection of proteins, the membranes were immunoblotted with rabbit polyclonal antibody specific for RIG-I, TLR3 (both from Thermo Fisher Scientific, NY, USA), total IRF7 (Abcam, Cambridge, MA, USA) and GAPDH (R&D Systems). The membrane signals were detected using horseradish peroxidase-labeled goat anti-rabbit IgG (Cell Signaling Technology, Beverly, MA, USA) and chemiluminescent reagents (Pierce Biotechnology, Rockford, IL, USA). Blots were viewed using the Syngene G:box Bioimaging System with GeneTools software (Syngene, Frederick, MD, USA) and quantified using ImageQuant software (BD/Molecular Dynamics, Bedford, MA, USA). 

### 2.7. Statistical Analysis

Where applicable, the data have been expressed as the means ± standard error of the mean (SEM). Statistical significance was determined by one-way ANOVA with Student–Newman–Keuls post hoc correction for multiple comparisons as appropriate. Significance was considered as *P* < 0.05.

## 3. Results

### 3.1. IRF7 Knockdown Inhibited Influenza-Initiated RIG-I and IFN Induction, but not IFN-β-Mediated RIG-I Induction in Human Bronchial BCi-NS1.1 Cells

**Figure 1:**
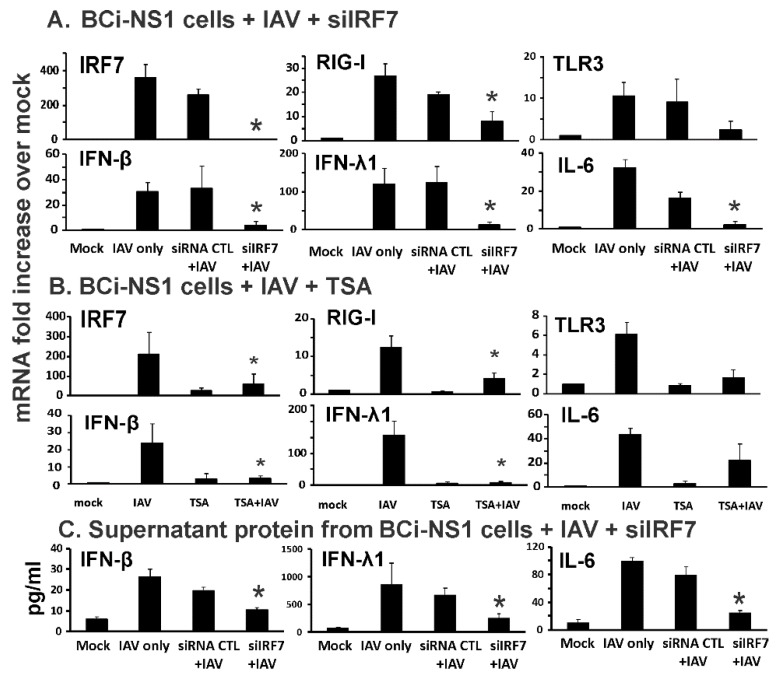
IRF7 knockdown by specific siRNA or chemical inhibition decreased influenza-induced innate responses in human airway BCi-NS1.1 basal cells. BCi-NS1.1 cells were transfected with IRF7 siRNA and cultured for 72 h (**A**) or these cells were treated with TSA (100 ng/mL), an ISGF3 inhibitor, for 16 h (**B**). After siRNA or TSA treatment, the cells were infected with IAV PR8 at the MOI of 0.2 for 24 h. Total RNA was extracted and mRNA expression was assessed by qRT-PCR. Transcript levels of mRNA were normalized relative to the constitutively expressed β-actin gene. (**C**) Cytokine protein levels released in the supernatant were assessed by ELISA. Data were expressed as the means ± SEM from three separate experiments. Statistical significance was determined by ANOVA. * denotes significant difference compared to data from the siRNA CTL+ IAV infected group, *p* < 0.05.

In lung epithelial cells, type I and III IFN induction by IAV is dependent on RIG-I and TLR3 activation. To examine the role of IRF7 induction in this process, we first examined the effect of IRF7 knockdown by specific siRNA on IRF7 mRNA induction by IAV PR8. IAV induced IRF7 mRNA 360 fold over the levels seen in mock-infected cells. Knockdown was successful as evidenced by almost complete (99%) inhibition of IRF7 mRNA induction in specific IRF7 siRNA (siIRF7)-treated cells relative to that seen in cells treated with siRNA control, when both were infected with IAV PR8 (Figure 1A). IAV infection of BCi-NS1.1 cells (Figure 1A) caused a 27 and 11 fold increase in RIG-I and TLR3 mRNA levels respectively, over the levels seen in mock-infected cells. RIG-I and TLR3 mRNA induction by IAV was decreased 53% and 74% in siIRF7 treated cells compared to control (CTL) siRNA treated cells. To further confirm the inhibition of RIG-I and TLR3 by IRF7 knockdown, we also assessed downstream cytokine induction by virus. IFN-β, IFN-λ1 and IL-6 mRNA induction by IAV was 31, 120 and 33 fold over mock treated cells, respectively. IFN-β, IFN-λ1 and IL-6 mRNA induction was decreased 88%, 81% and 88%, respectively, in siIRF7-treated virus-infected cells compared to siRNA CTL-treated virus-infected cells (Figure 1A). ELISA confirmed the inhibition of IFN-β, IFN-λ1 and IL-6 protein induction released in the supernatants from siIRF7-treated virus-infected cells (Figure 1C). Decreased IRF7, RIG-I and IL-6 expression upon siRNA CTL-treatment was observed but the difference was not significant. This may be due to off target effects of the siRNA CTL. To control for off target effects, we have calculated the decreased induction in virus-infected siRNA-treated cells compared not to the IAV only group, but to siRNA CTL-treated cells instead.

RIG-I is inducible by IFNs released from the same and neighboring IAV infected cells [20]. Secreted IFN-β proteins bind to its cognate cellular receptor and activate a specific JAK-STAT pathway. Following receptor activation by IFN, the transcription factors STAT1 and STAT2 are phosphorylated by Janus protein tyrosine kinases Jak1 and Tyk2 and released from their docking sites on the receptor. They then associate with IRF-9 and form the ISGF3 complex, which stimulates IFN-dependent gene transcription by binding to the IFN stimulated response element (ISRE) sequences located in the RIG-I promoter. We next used trichostatin A (TSA), an ISGF3 complex formation inhibitor, to examine if RIG-I induction in human BCi-NS1.1 cells is actually through the ISGF3 complex as has been reported in other model systems. As expected, we found that TSA significantly inhibited RIG-I, IRF7 and downstream cytokine induction by IAV in our human cell model (Figure 1B). This confirms that RIG-I mRNA induction by IAV requires assembly of the transcription factor complex ISGF3 in our human bronchial epithelial cells. 

Overall, the results demonstrate that *de novo* synthesis of IRF7 is critical in IFN and RIG-I mRNA induction during IAV infection in BCi-NS1.1 cells, and that RIG-I mRNA induction by IAV requires ISGF3 assembly. The results demonstrate, for the first time, that these events occur in human airway epithelial cells.

**Figure 2:**
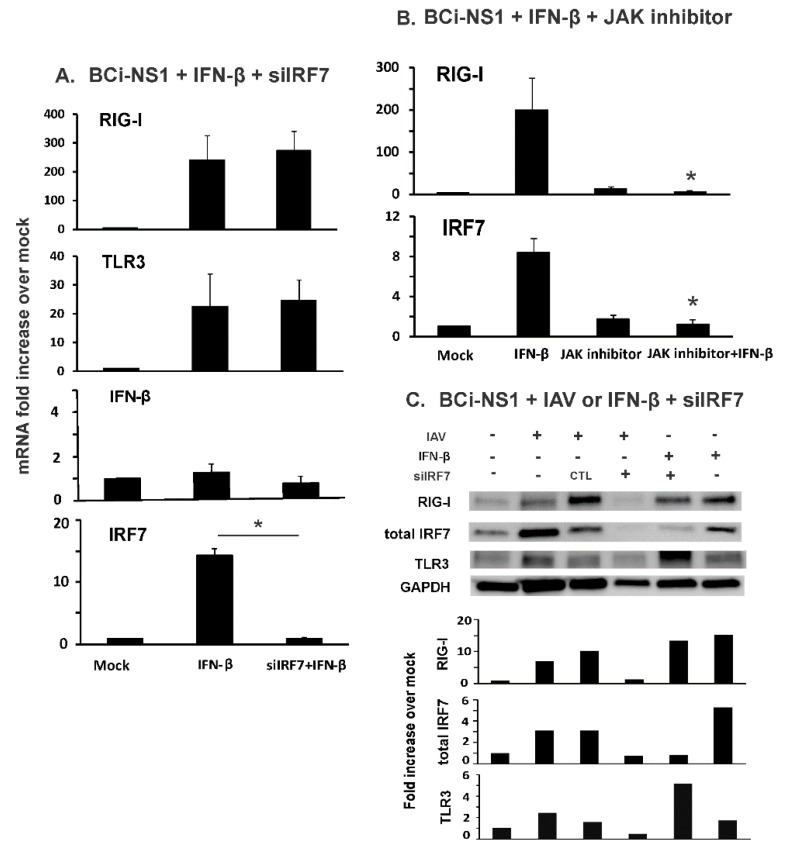
Knockdown of IRF7 did not inhibit IFN-β-mediated RIG-I and TLR3 induction in BCi-NS1.1 cells. BCi-NS1.1 cells were transfected with IRF7 siRNA for three days (**A**), or were treated with Ruxolitinib (2 µM), a JAK inhibitor, for 16 h (**B**). After the siRNA or TSA treatment, the cells were stimulated with 500 U/mL of IFN-β for 6 h. Total RNA was extracted and mRNA expression was assessed by qRT-PCR. Transcript levels of mRNA were normalized relative to the constitutively expressed β-actin gene. Data were expressed as the means ± SEM from three separate experiments. Statistical significance was determined by ANOVA. * denotes significant difference compared to data from IFN-β group, *p* < 0.05. (**C**) RIG-I, TLR3 and IRF7 proteins in BCi-NS1.1 cells were detected by immunoblotting. The immunoblot shown is representative of three separate experiments, with quantitation of that result depicted below the image.

To determine whether IRF7 is critical in the effects of first phase IFN induction on autocrine and paracrine amplification of RIG-I and TLR3 that occurs during viral infection, it is necessary to isolate induction of these receptors by IFN in the absence of virus. Therefore, we next examined RIG-I and TLR3 induction by IFN-β in siIRF7 treated, uninfected, BCi-NS1.1 cells. To mimic the effects of first phase IFN release only, these cells were stimulated with 500 U/ml of IFN-β for 6 h without IAV infection. As expected IFN-β treatment increased RIG-I mRNA 240-fold over mock treated cells (Figure 2A). It also induced TLR3 mRNA by 22 fold over controls. Furthermore, IFN-β treatment increased IRF7 mRNA 14-fold over mock treated cells, but did not significantly augment IFN-β mRNA levels, showing that first phase IFN effects are isolated using this method. Surprisingly, RIG-I and TLR3 mRNA induction was not affected by IRF7 knockdown during IFN-β treatment (Figure 2A). We also examined our cells with a JAK-STAT pathway inhibitor, Ruxolitinib, as an inhibition positive control since RIG-I and IRF7 mRNA induction by IFN-β requires JAK-STAT pathway activation [21]. As expected, the JAK-STAT pathway inhibitor significantly blocked RIG-I and IRF7 induction by IFN-β in BCi-NS1.1 cells (Figure 2B). We also determined protein expression levels by immunoblotting (Figure 2C). siIRF7 successfully inhibited IRF7 protein induction by IAV. Specifically, we found that IRF7 knockdown did not block RIG-I and TLR3 protein induction by IFN-β, but blocked RIG-I and TLR3 protein induction by IAV. 

Taken together, the data suggest that IRF7 does not mediate paracrine induction of RIG-I in non-infected cells caused by first phase IFN-β secreted from adjacent IAV infected cells. In contrast, IRF7 is important for full induction of IFN in IAV-infected BCi-NS1.1 cells. Therefore, the siIRF7 knockdown results suggest IRF7 indirectly modulates RIG-I and thus cytokine responses through inhibiting generation of IFN during influenza virus infection. 

### 3.2. MAVS, but not IRF3, Knockdown Inhibited Influenza-Initiated RIG-I and IFN Induction in Human Bronchial BCi-NS1.1 Cells

**Figure 3:**
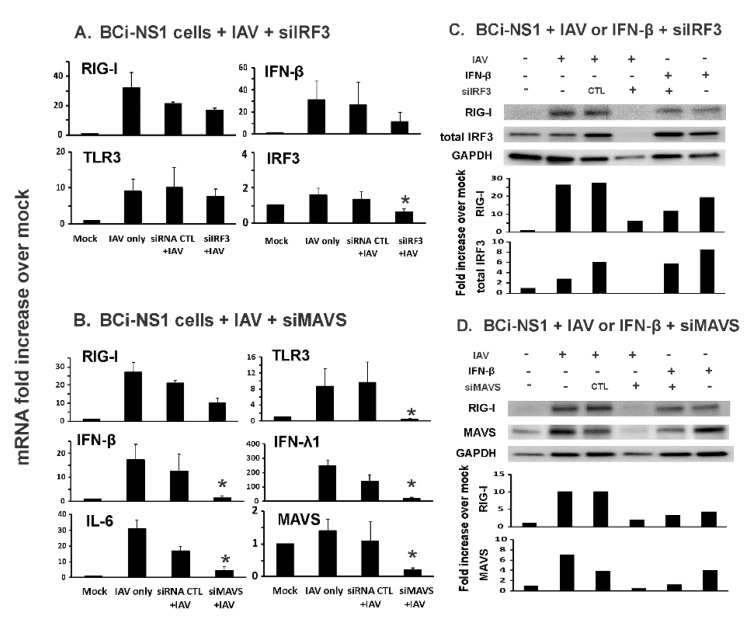
Knockdown of MAVS, but not IRF3, inhibited influenza-mediated RIG-I, TLR3 and cytokine mRNA induction in BCi-NS1.1 cells. The cells were first transfected with (**A** and **C**) IRF3 or (**B** and **D**) MAVS siRNA and cultured for 72 h and then infected with IAV PR8 (MOI = 0.2) for 24 h. Total RNA was extracted and mRNA expression was assessed by qRT-PCR (**A** and **B**). Data were expressed as the means ± SEM from three separate experiments. Statistical significance was determined by ANOVA. * denotes significant difference compared to data from the siRNA CTL+ IAV infected group, *p* < 0.05. (**C** and **D**) RIG-I, IRF3 and MAVS proteins in BCi-NS1.1 cells were detected by immunoblotting. The immunoblot shown is representative of three separate experiments, with quantitation of that result depicted below the image.

IRF3 is another transcription factor implicated in RIG-I mediated IFN induction, which could potentially induce RIG-I in an autocrine or paracrine fashion. We next tested whether this occurred in our system using siRNA and the relative roles of IRF3 and IRF7 in IFN induction. Notably, IRF3 mRNA induction by IAV (1.8-fold increase over mock, Figure 3A) was minimal when compared with IRF7 mRNA induction by IAV (370-fold increase over mock, Figure 1A). Furthermore, IRF3 knockdown reduced RIG-I and TLR3 mRNA induction by IAV to a lesser extent (20% and 22 %, respectively) than knockdown of IRF7 (59% and 73% inhibition of RIG-I and TLR3, respectively). Therefore, IRF3 is less involved in the induction of RIG-I and TLR3 by IAV than IRF7 in these cells (Figure 3A). 

IRF7 is phosphorylated and activated after IAV-induced binding of RIG-I to its adaptor, MAVS. If first-phase IFN synthesis in virus infected cells requires RIG-I, knockdown of MAVS will inhibit first phase IFN induction and subsequent RIG-I induction. We therefore used MAVS siRNA to knockdown the RIG-I adaptor in our BCi-NS1.1 human cell model. MAVS knockdown was confirmed as evidenced by the almost complete (99%) inhibition of MAVS mRNA induction in specific MAVS siRNA (siMAVS)-treated cells infected with IAV as compared to MAVS induction seen in IAV infected cells treated with siRNA control (Figure 3B). siMAVS treatment reduced RIG-I (63%) and TLR3 (96%) mRNA induction, as well as subsequent IFN-β (92%), IFN-λ1 (93%) and IL-6 (85%) induction by IAV in the cells (Figure 3B). Thus, the results demonstrate that first phase of IFN synthesis induced by IAV requires MAVS. We also confirmed the RIG-I mRNA expression change affected by siIRF3 and siMAVS at protein expression levels by immunoblotting (Figure 3C,D).

**Figure 4:**
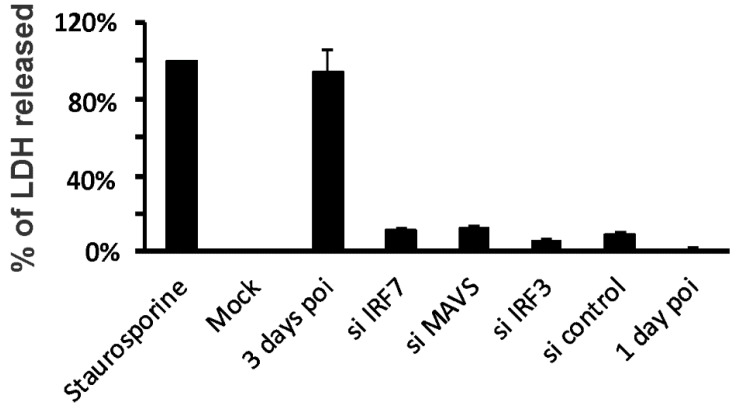
Effects of siRNA treatment and IAV infection on cell viability. BCi-NS1.1 were incubated with siRNA for 3 days. For IAV PR8 infected cells, BCi-NS1.1 were incubated with IAV (MOI = 0.2) for 1 or 3 days. Staurosporine (10 µM) treated cells were used as a positive control for cytotoxicity. LDH activity of the supernatant was measured by using the LDH-Cytotoxicity Assay Kit. Cytotoxicity is expressed as the percentage of supernatant released LDH to LDH released from Staurosporine treated cells. Results are shown as the Mean ± SEM from three separate experiments.

To determine whether the effects seen by siRNA knockdown could be due to non-specific cytotoxicity, we measured LDH release as a percentage of total LDH present (media + cells) during siRNA treatment. Cells were exposed to siIRF7, siMAVS, siIRF3 and siRNA control for 24 h, followed by the measurement of LDH in the media. Staurosporine was used as a positive control for cytotoxicity (Figure 4). Basal LDH release was low (mock). IAV infection caused BCi-NS1.1 cell death after 3 days, but not 1-day post-infection when the samples were collected. LDH levels of all siRNA treated cells and cells at 1-day post-infection were minor compared to the positive control. Thus, the effects in siIRF7- and siMAVS-treated cells is not due to non-specific cytotoxicity.

### 3.3. IRF7 Knockdown Inhibited Influenza-Initiated, but not IFN-β-Induced, RIG-I Induction in Human Alveolar Epithelial Cells

**Figure 5:**
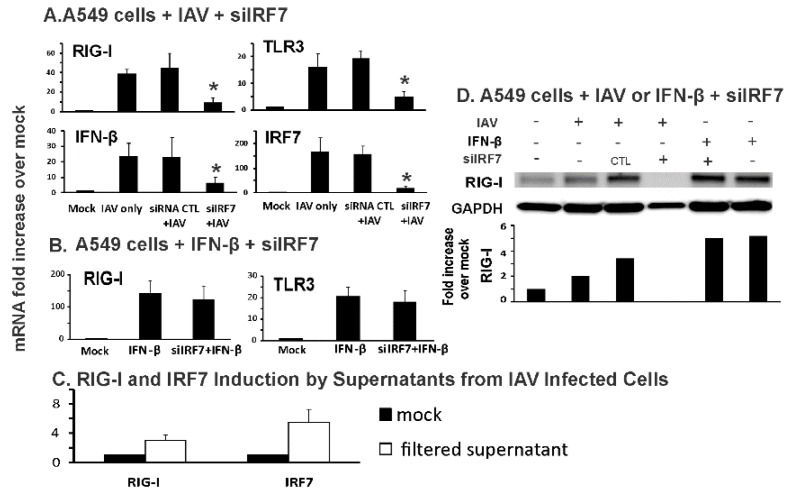
Knockdown of IRF7 inhibited influenza-mediated RIG-I induction and innate responses in human alveolar epithelial cells. A549 cells were transfected with IRF7 siRNA for three days. Then, the cells were infected with (**A**) IAV PR8 at the MOI of 0.2 for 24 h or (**B**) 500 U/mL of IFN-β for 6 h. Total RNA was extracted and mRNA expression was assessed by qRT-PCR. Data were expressed as the means ± SEM from three separate experiments. Statistical significance was determined by ANOVA. * denotes significant difference compared to data from the siRNA CTL + IAV infected group, *p* < 0.05. (**C**) Supernatants from IAV infected cells stimulated RIG-I/IRF7 expression in uninfected cells. Supernatants were collected from IAV infected cells after 24 h poi. Then the supernatants were filtered to remove IAV using a 100 K MW filter (Amicon). The filtered supernatants were added to A549 cells and changes in RIG-I/IRF7 mRNA were assessed by qRT-PCR. (**D**) RIG-I protein in A549 cells was detected by immunoblotting. The immunoblot shown is representative of three separate experiments, with quantitation of that result depicted below the image.

We extended our work to more distal epithelial cells in the lung, human alveolar epithelial cells. We used a common model for these cells, the A549 alveolar epithelial cell line. Infection with IAV PR8 resulted in a 40- and 15-fold increase in RIG-I and TLR3 mRNA levels, respectively, over mock-infected cells. RIG-I and TLR3 mRNA induction was decreased 80% and 76% in siIRF7 treated cells compared to control siRNA treated cells. Downstream IFN-β mRNA induction was decreased 72% in siIRF7-treated IAV-infected cells compared to control siRNA-treated cells (Figure 5A). In terms of the siIRF7 inhibition of its target, IRF7 mRNA induction by IAV was blocked 86% in the siIRF7-treated IAV-infected A549 cells (Figure 5A). 

As was the case for BCi-NS1.1 cells, when A549 cells were treated with IFN-β only, siIRF7 did not inhibit IFN-β-stimulated RIG-I and TLR3 induction (Figure 5B). We next examined if secreted IFN-β from IAV infected cells stimulated RIG-I/IRF7 expression in uninfected cells. Supernatants were collected from IAV infected cells after 24 h poi. Then the supernatants were filtered to remove IAV using a 100 K MW filter. Absence of IAV was confirmed using qRT-PCR for viral NP and matrix genes (not shown). The filtered supernatants were added to A549 cells. After 6 h, both RIG-I and IRF7 mRNA were induced by the supernatants (Figure 5C). We confirmed the effect of siRNA on RIG-I protein induction by IAV using Western blot (Figure 5D). In these cells, protein levels reflected mRNA expression levels, as there was no detectable RIG-I protein in IAV-infected A549 cells treated with siRNA for IRF7. In contrast, there was no inhibition of RIG-I protein induction by IFN-β with IRF7 siRNA treatment. The results suggested that, as we found in human bronchial BCi-NS1.1 cells, IRF7 knockdown inhibited influenza-initiated, but not IFN-β-induced, RIG-I induction in human alveolar AEC II cells. 

## 4. Discussion

The airway epithelium is strategically positioned at the interface with the environment, and thus plays a key role in the innate immune lung response to outside stimuli or infection [22]. The airway epithelium responds to IAV by increasing its production of mediators including cytokines and chemokines that can recruit and activate inflammatory cells [23]. Human airway basal cells (BC), a proliferating population of cells that reside in close proximity to the basement membrane, function as stem/progenitor cells of both the mouse and human airway epithelium and differentiate into the other specialized cell types during normal epithelial turnover and repair. In alveoli, the alveolar epithelia, comprised of type I alveolar epithelial cells (AEC I) and type II alveolar epithelial cells (AEC II), cover more than 99% of the internal surface area [24]. AEC II can replicate in the alveoli to replace damaged AEC I cells, playing a similar basal role as BC in bronchiole. 

Despite the evidence that BCs of the bronchioles and AEC II of the alveoli are stem cells, relatively little is known about their biology in innate immune responses to IAV. In this report, we examined the two most important human lung epithelial progenitor/stem cells, bronchial basal cells (BCi-NS1.1) and AEC II cells (A549). 

**Figure 6:**
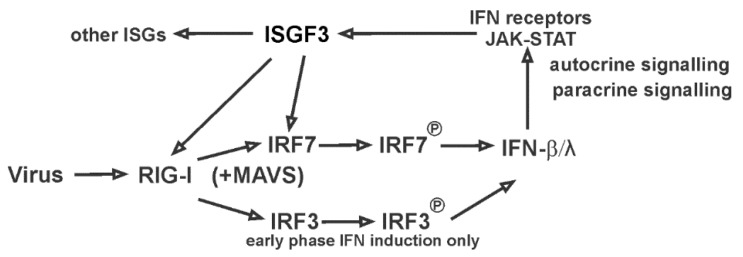
Proposed model of the RIG-I/IRF7/IFN positive-feedback amplification loop in human lung airway epithelial cells.

First, we found that the basal expression level, without virus stimulation, of RIG-I, TLR3 and IRF7 were very low in BCi-NS1.1 cells, and that IAV infection induced robust RIG-I, IRF7 and IFN expression (Figure 1). We then used exogenous IFN-β to examine the effects of produced IFN-β during the first phase of IFN induction on non-infected cells in the absence of IAV. IFN-β alone induced significant RIG-I, TLR3 and IRF7 expression (Figure 2). This is consistent with a model whereby secreted first phase IFN protein significantly elevates RIG-I, TLR3 and IRF proteins and turns these uninfected neighboring cells into an “immunoactive” state primed to respond to subsequent viral RNA detection. The second phase IFN induction is then amplified when IAV was detected in newly infected, IFN-stimulated cells. Thus, RIG-I, TLR3 and IRF7 expression is highly inducible by the first phase produced IFN. IRF7 is the master regulator of type-I IFN-dependent immune responses in vivo [25], but the role of IRF7 in human airway epithelial cell responses to IAV has not been previously investigated. Our data demonstrate that IRF7 controls the second-phase IFN amplification during influenza virus infection in these cells. The produced first-phase IFN stimulates RIG-I and IRF7 through JAK-STAT signaling and ISGF3 complex formation in a positive-feedback amplification loop during IAV infection in human lung epithelia (Figure 1 and Figure 2; model shown as Figure 6). IRF7 is not involved in RIG-I induction stimulated by the first-phase IFN-β in the neighboring cells, as RIG-I induction by IFN-β is not blocked by siIRF7 (Figure 2).

We found that knockdown of the transcription factor IRF3 only partially inhibited influenza-mediated RIG-I mRNA induction in BCi-NS1.1 cells. This result is consistent with the model proposed by Sato et al. [26], in that we showed that IRF7 must first be induced in response to IAV in order for early limited IFN production to occur. In another paper, the same group showed that the IRF3^−/−^ mouse embryonic fibroblasts produced IRF7 and IFN-β when infected with Newcastle disease virus, indicating both IRF7 and IFN-β induction is independent of IRF3 [20]. With regards to IRF3, it likely plays a role in the first phase of IFN induction, as there appeared to be modest inhibition of IFN-β by IAV (did not reach statistical significance, Figure 3). It is unlikely that IRF3 plays a significant role in the amplification of IFN-β induction, as IRF3 was minimally induced by IAV (Figure 3). The previous results from other laboratories examining the role of IRF7, IRF3 and RIG-I in IAV-induced IFN production was obtained from animal models. Our data are unique in that we demonstrate that IRF7, IRF3 and RIG-I play similar roles in human lung epithelial cells during IAV infection. We also show that MAVS is required for first-phase IFN production. Doubtless, IRF3 still plays an important role in IFN induction, even though it is not critical in the amplification phase. It is demonstrated that RNA synthesis and nuclear export are required for activation of the IFN induction cascade by IAV using IRF3 phosphorylation as a marker [27].

## 5. Conclusions

Our study demonstrates that the induction of two major PRRs, RIG-I and TLR3, in epithelial cells by IAV is through an IRF7-dependent positive amplification loop. IRF3 plays a minimum role in this amplification loop. Our current model is that, after cell entry, viral RNA is recognized by trace level RIG-I. RIG-I signaling induces, via MAVS adaptor protein, the activation of trace level transcription factors IRF3/7. Next, the IRFs induce type I IFNs (IFN-α and IFN-β), which are released outside the cells to stimulate the production of ISGs (including RIG-I and IRF7) in neighboring cells. In this way, the new produced RIG-I and IRF7 establish a positive feedback loop when they encounter more viruses (Figure 6). IRF7, but not IRF3, plays a central role in modulating the expression of RIG-I, TLR3 and antiviral responses during IAV infections. Whereas IRF3 is constitutively expressed, IRF3 is only important in early-phase IFN induction. IRF7 may therefore be an important molecular driver of the antiviral responses to IAV in human airway epithelial cells.
